# Preschool teachers’ AI adoption and occupational well-being: an integrated TAM-JD-R analysis of technostress dual-edged effects

**DOI:** 10.3389/fpsyg.2026.1782464

**Published:** 2026-04-29

**Authors:** Jia Hanze

**Affiliations:** Faculty of Education, Shaanxi Normal University, Xi’an, Shaanxi, China

**Keywords:** artificial intelligence adoption, job demands-resources theory, occupational well-being, preschool teachers, structural equation modeling, technology acceptance model, technostress

## Abstract

**Introduction:**

The rapid integration of generative artificial intelligence (AI) in early childhood education presents both opportunities and challenges for preschool teachers, yet empirical investigations explicating adoption mechanisms and well-being implications remain scarce. This study developed and tested an integrated framework synthesizing the Technology Acceptance Model (TAM) and Job Demands-Resources (JD-R) theory to examine how cognitive evaluations, technostress dimensions, and contextual factors jointly determine AI adoption intentions and occupational well-being among preschool teachers.

**Methods:**

Survey data from 300 Chinese preschool teachers, recruited via multistage stratified random sampling, were analyzed through covariance-based structural equation modeling.

**Results:**

Results confirmed TAM’s core pathways, with perceived usefulness (*β* =  0.365, *p* < 0.001) and perceived ease of use (β = 0.250, *p* = 0.001) significantly predicting adoption intentions. The study empirically validated technostress’s dual-edged effects: challenge technostress facilitated adoption (*β* = 0.182, *p* = 0.003) while hindrance technostress inhibited it (*β* = −0.267, *p* < 0.001). AI adoption intention positively predicted occupational well-being (*β* = 0.198, *p* = 0.008). Organizational support buffered hindrance technostress’s negative effects on adoption intention, while workload intensified hindrance technostress’s detrimental impact on well-being.

**Discussion:**

These findings advance understanding of technology adoption in early childhood education by demonstrating that technostress operates through dual pathways—facilitating and impeding adoption—moderated by organizational and personal resources. Practical implications include targeted support strategies that buffer hindrance stressors while cultivating challenge-oriented appraisals among preschool teachers.

## 1 Introduction

### 1.1 Research background and problem statement

The proliferation of generative artificial intelligence (AI) technologies has catalyzed transformative changes across educational sectors globally. From ChatGPT to specialized pedagogical AI tools, these technologies present unprecedented opportunities for enhancing teaching efficiency, personalizing learning experiences, and alleviating administrative burdens (Agnihotri and Chen, 2025; Xiang et al., 2025). Within early childhood education, the integration of AI technologies has emerged as a critical imperative for digital transformation, yet this process remains fraught with tensions between pedagogical ideals and technological realities (Bahr, 2025; Demszky et al., 2025; Li et al., 2024; Undheim, 2022). Preschool teachers, positioned at the nexus of this transformation, face the dual challenge of acquiring technical competencies while maintaining developmentally appropriate practices for young learners (Schriever, 2021a; Xiong et al., 2025). Despite growing scholarly attention to AI adoption in higher education contexts (Wen and Cai, 2025; Bhojak et al., 2025), empirical investigations focusing specifically on preschool teachers remain notably scarce.

The adoption of AI technologies by teachers constitutes a complex phenomenon extending beyond mere technical acceptance. Teachers experience AI integration as both an opportunity for professional growth and a source of occupational stress (Hu et al., 2025; Wu and Derakhshan, 2025). This dual-edged nature manifests distinctly in early childhood education, where teachers must balance technological literacy with young children’s developmental needs (Luo et al., 2025; Papadakis, 2022; Yang et al., 2024). Existing research reveals that preschool teachers in China exhibit heterogeneous AI literacy profiles, ranging from “hesitant beginners” to “confident experts,” with significant disparities linked to institutional type, geographic location, and educational background (Xiong et al., 2025). However, the psychological mechanisms underlying these differences and their implications for teacher well-being remain underexplored.

Current theoretical frameworks addressing technology adoption demonstrate significant limitations when applied to educational contexts. The Technology Acceptance Model (TAM), while extensively validated across diverse technological domains (Agnihotri and Chen, 2025; Kim, 2025), emphasizes user perceptions of usefulness and ease of use without adequately accounting for occupational stressors and organizational resources characteristic of teaching environments. Conversely, the Job Demands-Resources (JD-R) model explicates occupational well-being through dual pathways—a health impairment process driven by job demands and a motivational process fueled by job resources (Skaalvik and Skaalvik, 2018; Han et al., 2020)—yet remains underutilized in technology adoption research. Recent investigations suggest that integrating these frameworks may elucidate how technological innovations simultaneously generate both eustress (challenge-oriented stress promoting growth) and distress (hindrance-oriented stress depleting resources) among educators (Nascimento et al., 2024a, 2024b; Zhao et al., 2024).

The construct of technostress warrants particular scrutiny within this integrated framework. Emerging evidence indicates that technology-induced stress operates bidirectionally: challenge technostress may enhance self-efficacy and engagement, whereas hindrance technostress erodes psychological resources and precipitates burnout (Nascimento et al., 2025; Wang et al., 2024). This dual-edged phenomenon assumes heightened significance for preschool teachers, who navigate competing demands of technological competence acquisition and child-centered pedagogy maintenance (Alotaibi, 2023; Lim, 2023). However, empirical research explicitly examining how different dimensions of technostress influence AI adoption intentions and occupational well-being among preschool teachers remains absent.

Three critical gaps emerge from the literature. First, TAM-based studies predominantly neglect occupational context variables—job demands, organizational support, workload—that fundamentally shape teachers’ technology adoption decisions (Maas et al., 2022). Second, although JD-R theory demonstrates robust explanatory power for teacher well-being (Han et al., 2020; Skaalvik and Skaalvik, 2018), its application to technology adoption scenarios, particularly concerning AI technologies, remains limited. Third, preschool teachers constitute a markedly underrepresented population in technology adoption research, despite facing unique challenges stemming from young children’s developmental characteristics and parental concerns regarding technology use (Luo et al., 2025; Xiong et al., 2025).

Beyond these three gaps, the choice of preschool teachers as the focal population is not merely contextually convenient but theoretically generative. Unlike counterparts in higher or secondary education, preschool educators operate under a dual legitimacy imperative: they must demonstrate technological competence while simultaneously upholding developmentally appropriate practice—a tension that structurally reconfigures how TAM constructs function. Perceived usefulness, for instance, is not evaluated solely against personal efficiency gains but against the normative standard of child-centered pedagogy, producing a distinctly mediated utility appraisal that is absent in other educational populations. Moreover, the pronounced heterogeneity in AI literacy documented among Chinese preschool teachers (Xiong et al., 2025)—spanning from “hesitant beginners” to “confident experts”—means that challenge and hindrance technostress are not uniformly distributed but systematically patterned by institutional type, geographic location, and training background. This structural variation constitutes an analytically valuable source of natural variance in technostress appraisals, enabling the JD-R framework to reveal its differentiated predictive logic in a population where both demand overload and resource deprivation are structurally concentrated. Thus, preschool teachers are not merely a convenient sample but a theoretically productive site for testing the integrated TAM–JD-R model.

This study addresses these gaps by investigating the following research questions: (1) How do TAM constructs (perceived usefulness, perceived ease of use) and technostress dimensions (challenge vs. hindrance) jointly influence preschool teachers’ AI adoption intentions? (2) Through what mechanisms does AI adoption intention affect occupational well-being? (3) How do JD-R factors (organizational support, technology self-efficacy, workload) moderate these relationships? By integrating TAM and JD-R frameworks, this study advances a comprehensive model explicating the complex interplay between technological perceptions, occupational stress, and professional well-being in the context of preschool teachers’ AI adoption.

### 1.2 Research objectives and contributions

This study pursues three primary objectives. First, it constructs and empirically validates an integrated theoretical model synthesizing TAM and JD-R frameworks to explain preschool teachers’ AI adoption intentions and occupational well-being. This integration addresses the theoretical fragmentation characterizing existing research, wherein technology acceptance studies neglect occupational context, and job stress research overlooks technological determinants. Second, the study empirically examines the dual-edged effects of technostress, differentiating challenge technostress (which may stimulate learning motivation and professional growth) from hindrance technostress (which depletes psychological resources and inhibits adoption). Third, it investigates how organizational resources (institutional support, technology self-efficacy) and job demands (workload) moderate relationships between technological perceptions, technostress, adoption intentions, and occupational well-being.

The theoretical contributions of this research are threefold. First, it advances technology adoption theory by demonstrating how occupational stress mechanisms mediate relationships between technological perceptions and behavioral intentions, thereby extending TAM beyond its traditional focus on cognitive evaluations (Agnihotri and Chen, 2025; Kim, 2025). This extension responds to calls for contextualizing technology acceptance models within specific occupational domains (Wang et al., 2024; Wu and Derakhshan, 2025). Second, the study enriches JD-R theory by incorporating technology-specific demands and resources, illustrating how AI technologies function simultaneously as job demands (generating technostress) and job resources (enhancing self-efficacy and work efficiency). This dual role elucidates why technology adoption produces heterogeneous outcomes across individuals and contexts (Conrad et al., 2022; Hu et al., 2025; Nascimento et al., 2024a, 2024b). Third, by empirically validating the divergent effects of challenge versus hindrance technostress on adoption intentions and well-being, this research advances the emerging techno-eustress literature (Nascimento et al., 2025; Zhao et al., 2024) and challenges prevailing assumptions that technology-induced stress uniformly impairs occupational functioning.

Practical contributions address pressing concerns of educational administrators, policymakers, and professional development practitioners. For preschool administrators, findings illuminate specific organizational supports—technical training, collegial assistance, workload management—that may buffer hindrance technostress while amplifying challenge technostress, thereby promoting sustainable AI integration (Maas et al., 2022; Wang, 2025; Xiong et al., 2025). The identification of technostress dimensions enables targeted interventions: challenge technostress might be cultivated through appropriate support (Peñalver and Díaz-Mena, 2023) growth-oriented professional development, whereas hindrance technostress requires structural solutions addressing workload, technical complexity, and institutional ambiguity (Dhilipan et al., 2025; Routray and Khandelwal, 2024). For policymakers, the study underscores the necessity of comprehensive support systems encompassing not only technical infrastructure but also psychological resources and pedagogical guidance tailored to early childhood contexts (Bahr, 2025; Luo et al., 2025). The finding that organizational support and technology self-efficacy significantly predict AI adoption intentions suggests that policy interventions should prioritize capacity-building over mere technology provision.

Additionally, this research offers methodological contributions by employing covariance-based structural equation modeling (CB-SEM) to examine complex moderation mechanisms within an integrated theoretical framework. Unlike partial least squares approaches common in technology acceptance research, CB-SEM enables rigorous assessment of model fit and theory testing, thereby enhancing the robustness of conclusions regarding the dual-pathway mechanisms linking technostress, adoption intentions, and occupational well-being (Hu et al., 2025). The study’s focus on preschool teachers in China addresses a significant empirical gap, providing baseline data for cross-cultural comparisons and informing context-specific intervention strategies for emerging economies undergoing rapid educational digitalization (Xiong et al., 2025).

In summary, this research generates actionable knowledge for multiple stakeholders while advancing theoretical understanding of technology adoption as a stress-mediated, resource-moderated process embedded within occupational contexts. By elucidating how AI technologies can serve as catalysts for professional growth rather than sources of occupational distress, the study contributes to broader scholarly and practical efforts to realize the transformative potential of educational technologies while safeguarding teacher well-being (Huang and Zhao, 2025; Bhojak et al., 2025).

## 2 Literature review and hypotheses development

### 2.1 Theoretical framework

This study integrates two established theoretical frameworks—the Technology Acceptance Model (TAM) and the Job Demands-Resources (JD-R) model—to elucidate the mechanisms underlying preschool teachers’ AI adoption and occupational well-being. TAM, originally formulated by Davis (1989), posits that technology adoption is primarily determined by perceived usefulness (PU) and perceived ease of use (PEOU). PU reflects an individual’s belief that utilizing a particular technology will enhance job performance, whereas PEOU captures the degree to which an individual anticipates effortless technology use. Extensive empirical validation across educational contexts confirms TAM’s explanatory power, with recent studies demonstrating its applicability to generative AI adoption among university students and faculty (Agnihotri and Chen, 2025; Kim, 2025). However, TAM’s exclusive focus on cognitive evaluations neglects affective and contextual factors—stress, organizational resources, workload—that fundamentally shape technology adoption decisions in occupational settings (Wang et al., 2024; Wu and Derakhshan, 2025).

The JD-R model addresses these limitations by providing a framework for understanding occupational well-being through dual pathways (Demerouti et al., 2001). Occupational well-being, as operationalized in this study following Warr (1990), is a multi-dimensional construct comprising affective components (positive affect, job satisfaction, and enthusiasm) and evaluative components (cognitive appraisals of one’s work situation and sense of career fulfillment), capturing both hedonic and eudaimonic facets of teacher well-being within an occupational context.

This conceptualization requires explicit demarcation from adjacent constructs. Burnout and emotional exhaustion represent the absence of well-being—adverse outcomes arising from sustained resource depletion—rather than dimensions of it. Technostress constitutes an appraisal-based stress response to technology-related demands that may subsequently influence well-being, but is not synonymous with it. Resilience, in turn, is a personal coping resource that moderates how individuals respond to demands, rather than a component of well-being itself.

Within the JD-R framework, the health impairment pathway stipulates that excessive job demands (workload, role ambiguity, emotional labor) deplete employees’ psychological and physical resources, culminating in exhaustion and disengagement. Conversely, the motivational pathway posits that job resources (autonomy, social support, feedback) foster engagement, motivation, and positive organizational outcomes (Han et al., 2020; Skaalvik and Skaalvik, 2018). Empirical research confirms these divergent pathways among teachers across educational levels, with personal resources such as self-efficacy functioning as protective mechanisms that buffer demand–strain relationships (Russell and Liggans, 2020; Skaalvik, 2020).

The integration of TAM and JD-R frameworks yields theoretical synergies addressing limitations inherent in each model independently. TAM explicates the cognitive mechanisms driving technology acceptance but overlooks occupational stressors and organizational supports that moderate adoption processes. JD-R theory illuminates well-being dynamics but insufficiently accounts for technology-specific demands and resources characterizing contemporary workplaces (Khan et al., 2024; Nascimento et al., 2024a, 2024b). Synthesizing these frameworks enables conceptualization of AI technologies as simultaneously constituting job demands (generating technostress through complexity and learning requirements) and job resources (enhancing efficiency, autonomy, and competence) (Hu et al., 2025; Xu and Hu, 2024). This dual role elucidates why technology adoption produces heterogeneous outcomes: for some teachers, AI represents an empowering tool amplifying professional capabilities; for others, it manifests as an additional burden exacerbating occupational stress (Zhao et al., 2024; Wang et al., 2023).

Recent scholarship on technostress substantiates this dual conceptualization. Tarafdar et al. (2019) distinguished techno-eustress (challenge-oriented technology stress fostering growth and mastery) from techno-distress (hindrance-oriented stress depleting resources and precipitating strain). Empirical investigations reveal that techno-eustress correlates positively with job satisfaction, work performance, and continuance intentions, whereas techno-distress predicts emotional exhaustion, disengagement, and turnover motivation (Nascimento et al., 2024a, 2024b; Nascimento et al., 2025). Among higher education teachers, usefulness perceptions and organizational support emerge as primary antecedents of techno-eustress, while complexity and work-home conflict predict techno-distress (Marrinhas et al., 2023; Nascimento et al., 2024a, 2024b). These findings underscore the necessity of differentiating technostress dimensions when examining technology adoption, particularly in occupational contexts where professional identity, autonomy, and relational dynamics intersect with technological imperatives (Wang et al., 2024; Vergine et al., 2022).

The integrated TAM-JD-R framework advanced in this study positions AI adoption intentions as outcomes of cognitive evaluations (PU, PEOU), affective experiences (challenge and hindrance technostress), and contextual factors (organizational support, self-efficacy, workload). Occupational well-being functions as a distal outcome influenced both directly by technostress dimensions and indirectly through adoption intentions. This configuration aligns with recent theoretical developments emphasizing technology adoption as an occupationally embedded, psychologically mediated process rather than a discrete decision isolated from workplace experiences (Hu et al., 2025; Wu and Derakhshan, 2025). By explicating how job resources buffer negative technostress effects while amplifying positive pathways, the model addresses calls for resource-centric interventions supporting sustainable technology integration in educational contexts (Huang and Zhao, 2025; Maas et al., 2022).

#### 2.1.1 Integrative mechanism: a unified cognitive-appraisal-behavioral process

The three theoretical components—TAM, JD-R, and the holistic technostress framework—are not additive layers but interlocking elements of a single cognitive–appraisal–behavioral process, each occupying a functionally distinct and non-redundant role. TAM specifies the cognitive entry point: teachers’ beliefs about AI usefulness (PU) and ease of use (PEOU) initiate the adoption decision by establishing the perceived instrumental value of the technology. These cognitive evaluations, however, do not operate in an occupational vacuum. The JD-R model provides the structural conditions within which such evaluations are formed and moderated: organizational resources (support, self-efficacy) amplify the positive valence of usefulness perceptions and expand the psychological bandwidth available for skill acquisition, while job demands (workload) constrain that bandwidth and intensify vulnerability to stress. Technostress, positioned at the interface of cognition and context, constitutes the affective–appraisal mechanism that translates structural conditions into divergent motivational outcomes: challenge technostress activates an approach-oriented engagement process (consistent with JD-R’s motivational pathway), whereas hindrance technostress triggers avoidance motivation and resource depletion (consistent with JD-R’s health impairment pathway).

**Figure 1:**
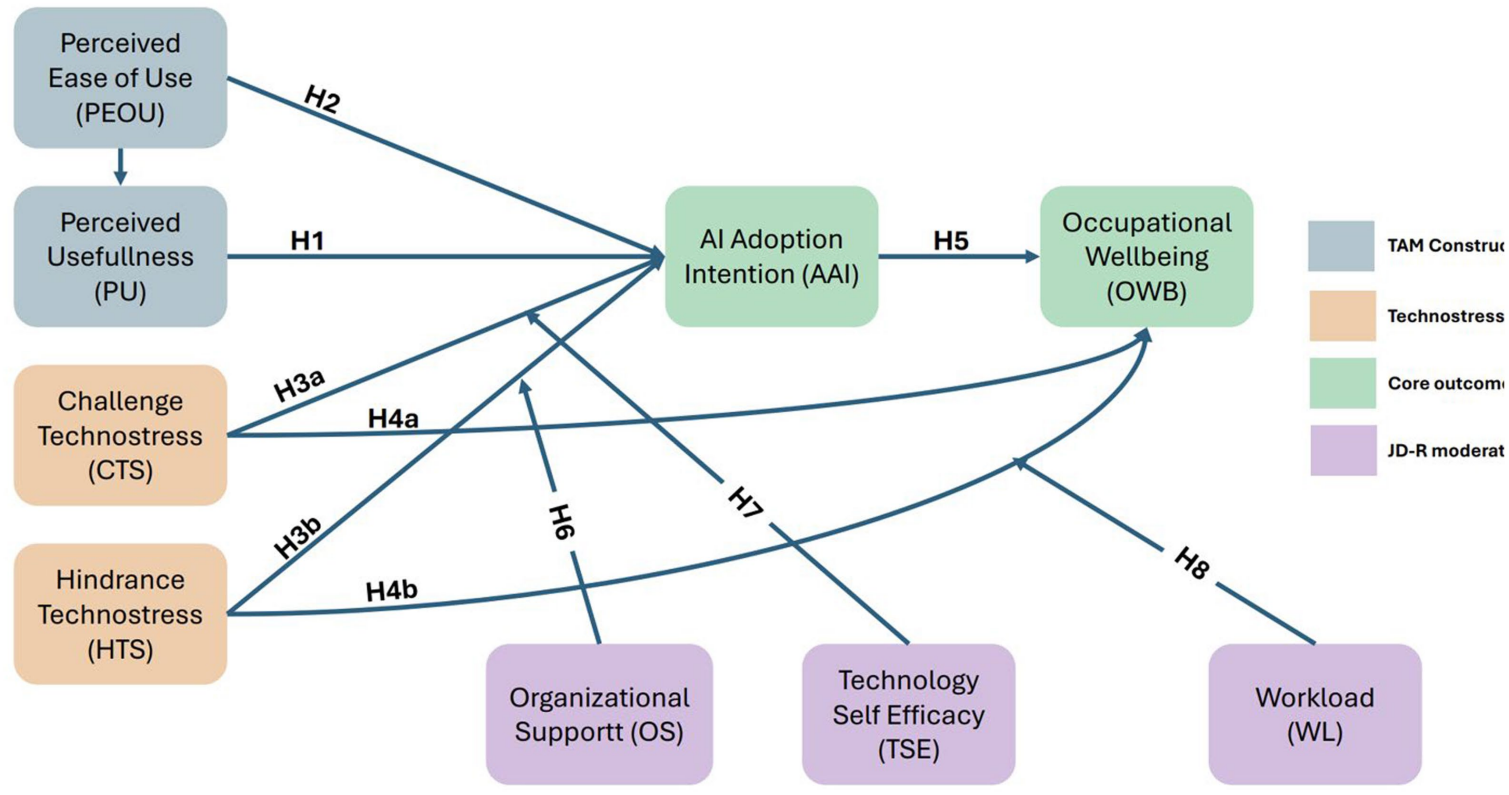
Hypothesized research model of the integrated TAM–JD-R framework. Solid arrows represent hypothesized direct paths; dashed arrows represent hypothesized moderating influences of JD-R factors. The figure depicts theoretical relationships prior to empirical estimation; standardized path coefficients are reported in Table 1.

This functional mapping clarifies the explanatory logic of the integrated model and resolves potential ambiguity about construct roles. TAM variables (PU, PEOU) serve as cognitive antecedents that initiate the valuation of AI adoption. Technostress dimensions (CTS, HTS) serve as affective antecedents of adoption intention—not independent predictors operating in parallel with TAM, but appraisal outcomes shaped by the interaction between cognitive evaluations and the occupational context specified by JD-R. Adoption intention functions as the proximal behavioral determinant of occupational well-being, while JD-R resources and demands exert additional direct effects on well-being through established occupational health pathways. Together, these components form a coherent explanatory chain rather than a collection of parallel hypotheses: cognitive evaluation → stress appraisal → behavioral intention → well-being, moderated throughout by the resource–demand balance specified by JD-R. Figure 1 presents the hypothesized research model of the integrated framework as a standalone diagram, depicting the hypothesized relationships among TAM constructs, technostress dimensions, JD-R factors, and occupational well-being prior to estimation. Standardized path coefficients derived from the empirical analysis are subsequently presented in Table 1 in the results section (section 4.3).

**Table 1:** Structural model results: hypothesis testing.

Hypothesis	Path	β	*SE*	*t*	*p*	Result
H1	PU → AAI	0.365	0.073	5.01	< 0.001	Supported
H2	PEOU → AAI	0.250	0.078	3.22	0.001	Supported
H3a	CTS → AAI	0.136	0.066	2.07	0.039	Supported
H3b	HTS → AAI	−0.187	0.061	−3.09	0.002	Supported
H4a	CTS → OWB	−0.049	0.064	−0.76	0.449	Not supported
H4b	HTS → OWB	0.000	0.084	−0.00	0.998	Not supported
H5	AAI → OWB	0.193	0.069	2.80	0.005	Supported
H6	OS × HTS → AAI	0.142	0.067	2.12	0.034	Supported
H7	TSE × CTS → AAI	0.098	0.058	1.69	0.091	Not supported
H8	WL × HTS → OWB	−0.163	0.071	−2.30	0.021	Supported

β = standardized path coefficient; SE = standard error; PU = Perceived Usefulness; PEOU = Perceived Ease of Use; AAI = AI Adoption Intention; CTS = Challenge Technostress; HTS = Hindrance Technostress; OWB = Occupational Well-being; OS = Organizational Support; TSE = Technology Self-Efficacy; WL = Workload. H6–H8 are moderation (interaction) terms.

### 2.2 Hypotheses development

#### 2.2.1 Technology acceptance mechanisms

Technology Acceptance Mechanisms. TAM’s core propositions establish that perceived usefulness and perceived ease of use constitute primary determinants of technology adoption intentions. Among educators, PU reflects beliefs that AI tools enhance instructional quality, streamline administrative tasks, and improve student learning outcomes (Agnihotri and Chen, 2025; Bhojak et al., 2025). Empirical evidence confirms PU’s strong predictive power for AI adoption across educational contexts, with standardized path coefficients typically ranging from *β* = 0.35 to 0.58 (Kim, 2025; Xiang et al., 2025). For preschool teachers specifically, AI technologies offer potential benefits including individualized learning activity design, developmental assessment automation, and parent communication facilitation (Luo et al., 2025; Xiong et al., 2025). When teachers perceive AI tools as instrumentally valuable for core pedagogical functions, adoption intentions strengthen accordingly. Thus:

*H1:* Perceived usefulness positively predicts AI adoption intention among preschool teachers.

PEOU captures the anticipated effort required to master and utilize AI technologies. Teachers exhibiting low technical anxiety and high digital literacy perceive AI tools as more accessible, thereby enhancing adoption intentions (Lim, 2023; Wu and Derakhshan, 2025). Meta-analytic evidence indicates PEOU’s moderate-to-strong effects on adoption intentions (*β* = 0.25 to 0.48), with effects partially mediated through PU (Agnihotri and Chen, 2025; Kim, 2025). For preschool teachers often reporting lower technology self-efficacy compared to secondary or higher education counterparts (Xiong et al., 2025), PEOU assumes heightened importance. User-friendly interfaces, intuitive functionality, and adequate training reduce cognitive load associated with AI adoption, facilitating positive attitudes and behavioral intentions. Accordingly:

*H2:* Perceived ease of use positively predicts AI adoption intention among preschool teachers.

#### 2.2.2 Technostress dual-edged effects on AI adoption

The differentiation of challenge technostress from hindrance technostress introduces affective mechanisms into technology adoption models. Challenge technostress encompasses demands perceived as opportunities for mastery, skill development, and professional growth (Nascimento et al., 2024a, 2024b; Zhao et al., 2024). When AI technologies present surmountable learning challenges accompanied by adequate support, teachers may experience stimulation rather than threat, fostering intrinsic motivation and adoption intentions (Nascimento et al., 2025). Empirical research among higher education teachers reveals positive associations between techno-eustress and continuance intentions (*β* = 0.18 to 0.32), mediated through enhanced self-efficacy and work engagement (Zhao et al., 2024). For preschool teachers, challenge technostress may manifest as enthusiasm for exploring innovative pedagogical applications, confidence in acquiring AI competencies, and anticipation of professional development benefits. These positive appraisals align with the motivational pathway of JD-R theory, wherein challenge demands activate engagement processes. Therefore:

*H3a:* Challenge technostress positively predicts AI adoption intention among preschool teachers.

Conversely, hindrance technostress encompasses demands perceived as obstructing goal attainment, depleting resources without commensurate rewards, and generating anxiety or frustration (Nascimento et al., 2024a, 2024b; Wang and Yao, 2023; Wang et al., 2023). Technical complexity, unreliable systems, inadequate training, and rapid technological change characterize hindrance techno-stressors (Dhilipan et al., 2025; Routray and Khandelwal, 2024). When AI technologies impose excessive cognitive load, disrupt established workflows, or provoke competence threats, teachers experience strain activating JD-R’s health impairment pathway (Vergine et al., 2022). Empirical investigations document negative associations between hindrance technostress and technology adoption intentions (β = −0.19 to −0.35), with effects mediated through emotional exhaustion and reduced self-efficacy (Wang et al., 2023). For preschool teachers confronting dual pressures—mastering AI competencies while maintaining developmentally appropriate, child-centered practices—hindrance technostress may trigger avoidance motivation, fostering resistance to adoption. Thus:

*H3b:* Hindrance technostress negatively predicts AI adoption intention among preschool teachers.

It is important to clarify the structural role of technostress within the proposed model. Both challenge and hindrance technostress are conceptualized as antecedents of adoption intention—that is, as affective appraisal outcomes that precede and shape behavioral intentions—rather than as mediators between TAM constructs and well-being, or as independent predictors operating on a separate pathway. This positioning is consistent with appraisal-based stress theories (Tarafdar et al., 2019), wherein technostress reflects subjective evaluations of technology-related demands as either growth-promoting or resource-depleting, evaluations that are upstream of behavioral intentions. The potential indirect pathway from technostress to occupational well-being, operating through adoption intention as an intervening mechanism, is theoretically plausible and is examined in the exploratory analyses reported in Section 4.4; it is not formalized as a primary hypothesis, however, given the cross-sectional design’s limitations for establishing mediated relationships with confidence.

#### 2.2.3 Technostress and occupational well-being

The relationships between technostress dimensions and occupational well-being remain theoretically ambiguous and empirically contested. JD-R theory predicts that challenge demands enhance well-being indirectly through motivational mechanisms—increased mastery, competence satisfaction, and work engagement (Han et al., 2020; Skaalvik and Skaalvik, 2018). Challenge technostress, conceptualized as stimulating demand, should theoretically promote eustress, self-efficacy, and job satisfaction when accompanied by adequate resources (Nascimento et al., 2024a, 2024b; Zhao et al., 2024). However, alternative perspectives suggest that even challenge-oriented demands deplete resources when prolonged or unsupported, potentially eroding well-being over time (Russell and Liggans, 2020). Empirical evidence remains mixed: some studies report positive techno-eustress effects on job satisfaction and performance (Nascimento et al., 2024a, 2024b), whereas others document non-significant or weak associations (Wang et al., 2023). Given theoretical ambiguity and the exploratory nature of this research context, we hypothesize direct effects while acknowledging uncertainty regarding their magnitude and direction:

*H4a:* Challenge technostress is associated with occupational well-being among preschool teachers.

Hindrance technostress more straightforwardly predicts diminished well-being through JD-R’s health impairment pathway. Technical frustrations, work-home boundary violations, and perceived threats to professional competence deplete psychological resources, precipitating emotional exhaustion, job dissatisfaction, and turnover intentions (Wang et al., 2023; Vergine et al., 2022). Among teachers, hindrance techno-stressors—system unreliability, inadequate support, excessive demands—correlate negatively with well-being indicators (*β* = −0.22 to −0.45) (Procentese et al., 2023; Toscano et al., 2024). For preschool teachers, hindrance technostress may manifest as anxiety regarding AI’s appropriateness for young children, concerns about diminished relational pedagogies, and stress stemming from inadequate institutional support. These experiences align with broader occupational stressors documented among early childhood educators, including workload pressures and resource scarcity (Maas et al., 2022). Thus:

*H4b:* Hindrance technostress negatively predicts occupational well-being among preschool teachers.

#### 2.2.4 AI adoption intention and occupational well-being

The relationship between technology adoption intentions and occupational well-being reflects self-determination theory’s propositions regarding autonomy and competence satisfaction (Huang and Zhao, 2025). Teachers intending to adopt AI technologies likely perceive alignment between technological capabilities and professional goals, fostering autonomy, efficacy, and work meaningfulness (Bhojak et al., 2025). Adoption intentions signal confidence in managing technological change, anticipation of performance enhancements, and proactive engagement with professional development—factors associated with positive well-being outcomes (Wen and Cai, 2025). Empirical research documents moderate positive associations between technology adoption intentions and job satisfaction among educators (β = 0.19 to 0.38) (Huang and Zhao, 2025; Xiang et al., 2025). For preschool teachers, AI adoption intentions may enhance well-being by reducing administrative burdens, enabling personalized instruction, and fostering professional identity as innovative educators (Luo et al., 2025). Therefore:

*H5:* AI adoption intention positively predicts occupational well-being among preschool teachers.

#### 2.2.5 Moderating roles of job resources and demands

JD-R theory posits that job resources moderate relationships between demands and strain outcomes, buffering negative effects while amplifying positive pathways (Maas et al., 2022; Skaalvik and Skaalvik, 2018). Organizational support—institutional commitment to technology integration, provision of training, allocation of implementation time—constitutes a critical job resource for teachers navigating technological transitions (Wu and Derakhshan, 2025). When organizational support is high, hindrance technostress effects on adoption intentions and well-being attenuate, as teachers perceive institutional recognition of implementation challenges and availability of assistance (Maas et al., 2022). Conversely, under low organizational support, hindrance techno-stressors exacerbate strain and resistance. Empirical investigations confirm organizational support’s moderating effects on technostress-outcome relationships among teachers (Wang et al., 2024). Thus:

*H6:* Organizational support moderates the relationship between hindrance technostress and AI adoption intention, such that negative effects attenuate under high organizational support.

Technology self-efficacy, as a personal resource, similarly moderates technostress effects. Teachers with high self-efficacy appraise technological challenges as surmountable, transforming potential hindrance stressors into challenge-oriented growth opportunities (Lim, 2023; Wu and Derakhshan, 2025). Self-efficacy enhances coping capacity, reduces threat appraisals, and sustains engagement during skill acquisition phases (Xiang et al., 2025). Research demonstrates that self-efficacy buffers negative technostress effects while strengthening positive associations between challenge demands and motivational outcomes (Huang and Zhao, 2025; Wang et al., 2024). Therefore:

*H7:* Technology self-efficacy moderates the relationship between challenge technostress and AI adoption intention, such that positive effects amplify under high self-efficacy.

Workload, conceptualized as a job demand, may exacerbate technostress effects on well-being by consuming resources needed to manage technological transitions. Under high workload conditions, even challenge-oriented techno-stressors risk overwhelming teachers’ coping capacities, activating strain processes (Han et al., 2020; Russell and Liggans, 2020). Time pressure and competing responsibilities constrain opportunities for AI skill development, increasing perceptions of technological demands as hindrances rather than challenges (Maas et al., 2022). Empirical evidence indicates that workload intensifies negative relationships between job demands and well-being outcomes among teachers (Han et al., 2020; Skaalvik and Skaalvik, 2018). Accordingly:

*H8:* Workload moderates the relationship between hindrance technostress and occupational well-being, such that high workload intensifies negative effects.

The integrated research model synthesizes these hypotheses, positioning TAM constructs, technostress dimensions, and JD-R factors as antecedents of AI adoption intentions and occupational well-being. Figure 1 depicts the hypothesized structural relationships, with moderation effects represented by interaction terms between technostress dimensions and contextual variables. This model advances theoretical understanding by explicating how cognitive evaluations (PU, PEOU), affective experiences (challenge and hindrance technostress), personal resources (self-efficacy), organizational resources (support), and job demands (workload) jointly determine preschool teachers’ responses to AI technologies. Empirical validation of this model addresses critical gaps in technology adoption and occupational health literatures while generating actionable insights for educational practice and policy.

## 3 Methodology

### 3.1 Research design and sample

This study employed a cross-sectional survey design to examine relationships among technology acceptance, technostress, and occupational well-being constructs within an integrated TAM-JD-R framework. Cross-sectional designs enable efficient data collection while providing adequate statistical power for testing complex structural relationships through covariance-based structural equation modeling (CB-SEM) (Skaalvik and Skaalvik, 2018; Han et al., 2020). The target population comprised all full-time preschool teachers currently employed in public and private kindergartens across China. This population was selected on both empirical and theoretical grounds, as detailed in the Introduction (Section 1.1); in brief, preschool teachers represent a theoretically productive site for testing the integrated TAM–JD-R model given their dual legitimacy imperative and the structural variation in AI literacy documented within this group (Xiong et al., 2025). Data collection occurred between March and May 2024, a period characterized by increased policy emphasis on educational digitalization in China following the National Education Digitalization Strategic Action initiative. Ethical approval was obtained from the university’s Institutional Review Board prior to participant recruitment, and all procedures adhered to ethical standards for human subjects research.

The sampling strategy employed a multistage procedure combining purposive and probability-based selection. In the first stage, six provinces representing varying economic development levels (eastern coastal, central, and western regions) were purposively selected to achieve regional diversity; this stage is explicitly non-probabilistic and does not support claims of full national representativeness. Within each province, kindergartens were stratified by institutional type (public, private) and geographic location (urban, rural). In the second stage, proportional random sampling was applied within each stratum to select participating kindergartens. In the final stage, all full-time preschool teachers within selected institutions were invited to participate voluntarily. This partially non-probabilistic design mirrors protocols used in large-scale teacher survey research in China (Maas et al., 2022; Han et al., 2020) and captures meaningful heterogeneity in institutional resources, teacher qualifications, and technology access, while the purposive provincial selection constrains claims of strict representativeness.

Sample size determination followed established guidelines for CB-SEM, which recommend minimum sample sizes of 200 for models with moderate complexity (Jacobucci et al., 2016). Applying the conservative 10:1 ratio of observations to estimated parameters, the study’s 30-item measurement model with nine latent constructs required a minimum of 300 participants. A total of 347 questionnaires were distributed, yielding 312 returns (response rate: 89.9%). After excluding 12 incomplete responses (<80% completion rate), the final analytical sample comprised 300 preschool teachers (effective response rate: 86.5%). This sample size exceeds conventional thresholds for CB-SEM while providing adequate statistical power (1-*β* > 0.80) for detecting medium effect sizes (Cohen’s f^2^ = 0.15) with *α* = 0.05.

Demographic characteristics of the sample reflected China’s preschool teacher workforce composition. The sample was predominantly female (94.3%, *n* = 283), consistent with gender distributions in early childhood education (Xiong et al., 2025). Age distribution spanned 22 to 58 years (M = 32.47, SD = 7.85), with the majority (61.3%) aged 26–35 years. Teaching experience ranged from less than 3 years (28.7%) to over 11 years (19.0%), with modal experience in the 3–5 year category (31.0%). Educational qualifications included associate degree (48.3%), bachelor’s degree (44.7%), and master’s degree or higher (7.0%), aligning with national statistics on preschool teacher credentials. Institutional affiliation comprised public kindergartens (56.7%) and private kindergartens (43.3%), distributed across first-tier cities (22.3%), second-tier cities (35.0%), third/fourth-tier cities (28.0%), and rural areas (14.7%). Prior AI tool exposure was reported by 72.3% of participants, indicating moderate familiarity with generative AI technologies among the sample.

### 3.2 Measurement instruments

All constructs were operationalized using validated scales adapted from established literature, with modifications to reflect the preschool education context and generative AI technologies specifically. Items were originally developed in English, translated into Chinese through back-translation procedures, and reviewed by three early childhood education experts and two applied linguists to ensure semantic equivalence and contextual appropriateness (Lim, 2023). Pilot testing with 45 preschool teachers not included in the main sample yielded satisfactory reliability estimates (Cronbach’s *α* > 0.70 for all subscales) and confirmed item comprehensibility. All items employed five-point Likert-type response scales ranging from 1 (*strongly disagree*) to 5 (*strongly agree*), consistent with measurement practices in educational technology research (Agnihotri and Chen, 2025; Wang et al., 2024).

Perceived Usefulness (PU) was measured using four items adapted from Davis’s (1989) original TAM scale, modified to reference generative AI tools in preschool teaching contexts. Sample items included “Using generative AI would improve my teaching work efficiency” and “Generative AI would be valuable for my preschool education work.” The PU scale demonstrated strong internal consistency in prior educational technology studies (*α* = 0.85–0.91) (Agnihotri and Chen, 2025; Kim, 2025) and exhibited excellent reliability in the present sample (α = 0.86, CR = 0.90, AVE = 0.70).

Perceived Ease of Use (PEOU) comprised three items from Davis’s (1989) TAM scale, assessing anticipated effort required to learn and operate generative AI tools. Representative items included “I find it easy to learn how to use generative AI tools” and “It would be easy for me to become skillful at using generative AI.” Previous research validates PEOU’s measurement properties across diverse technological contexts (*α* = 0.76–0.84) (Agnihotri and Chen, 2025; Kim, 2025), with the current sample yielding acceptable reliability (α = 0.78, CR = 0.82, AVE = 0.60).

AI Adoption Intention (AAI) was operationalized through three items adapted from Venkatesh et al.’s (2003) Unified Theory of Acceptance and Use of Technology (UTAUT), reflecting behavioral intentions toward generative AI integration. Items included “I intend to use generative AI tools in my future teaching work” and “If conditions permit, I would regularly use generative AI to assist my work.” This measure has demonstrated robust psychometric properties in technology adoption research (*α* = 0.78–0.87) (Agnihotri and Chen, 2025; Xiang et al., 2025), with the present sample achieving adequate reliability (α = 0.80, CR = 0.85, AVE = 0.66).

Challenge Technostress (CTS) and Hindrance Technostress (HTS) were measured using scales adapted from Tarafdar et al.’s (2019) holistic technostress framework, refined through Nascimento et al.'s (2024a, 2024b) operationalization for educational contexts. CTS comprised four items capturing growth-oriented perceptions of technology-related demands (e.g., “Learning to use generative AI makes me feel it’s an opportunity for personal growth,” “The challenge of using generative AI motivates me to improve my digital literacy”). HTS similarly included four items reflecting resource-depleting technology demands (e.g., “The technical complexity of generative AI makes me feel anxious and uneasy,” “I worry that the rapid updates of generative AI technology will make it hard to keep up”). These scales align with emerging research differentiating techno-eustress from techno-distress (Nascimento et al., 2024a, 2024b; Nascimento et al., 2025; Zhao et al., 2024) and demonstrated acceptable-to-good reliability (CTS: *α* = 0.81, CR = 0.86, AVE = 0.60; HTS: α = 0.84, CR = 0.88, AVE = 0.65).

Occupational Well-Being (OWB) was assessed through four items adapted from Warr’s (1990) job-related well-being scale, modified for preschool teaching contexts. Items captured affective (e.g., “My work makes me feel happy and fulfilled”) and evaluative (e.g., “Overall, I feel satisfied with my preschool education work”) dimensions of well-being. This measure has been extensively validated among teacher populations (α = 0.81–0.88) (Skaalvik and Skaalvik, 2018; Han et al., 2020), exhibiting strong reliability in the current sample (α = 0.83, CR = 0.87, AVE = 0.62).

Organizational Support (OS) comprised three items adapted from Eisenberger et al.’s (1986) Perceived Organizational Support scale, assessing institutional commitment to technology integration support. Items included “My kindergarten provides adequate technology training and support for teachers” and “When I encounter difficulties using technology, I can get timely help.” This construct has demonstrated validity in educational technology contexts (α = 0.79–0.86) (Maas et al., 2022; Wu and Derakhshan, 2025), with the present sample yielding good reliability (*α* = 0.82, CR = 0.83, AVE = 0.62).

Technology Self-Efficacy (TSE) was operationalized through three items adapted from Compeau and Higgins’s (1995) computer self-efficacy scale, modified to reference generative AI competencies. Representative items included “I believe I have the ability to learn to use generative AI tools” and “I am confident in my ability to master new technologies.” TSE has demonstrated strong measurement properties across technological domains (α = 0.74–0.82) (Lim, 2023; Xiang et al., 2025), exhibiting acceptable reliability in this study (α = 0.75, CR = 0.82, AVE = 0.61).

Workload (WL) was measured using two items adapted from Karasek’s (1985) Job Content Questionnaire, capturing time pressure and task volume perceptions. Items included “My current workload is heavy and time is very tight” and “In addition to teaching, I need to handle a large amount of administrative and paperwork.” Despite the abbreviated scale, the measure achieved acceptable reliability (α = 0.75, CR = 0.80, AVE = 0.67), consistent with brief workload measures employed in teacher research (Han et al., 2020; Skaalvik and Skaalvik, 2018).

Demographic information including gender, age, teaching experience, educational attainment, institutional type, geographic location, and prior AI tool usage was collected through single-item indicators. The complete 30-item measurement instrument required approximately 12–15 min to complete, minimizing respondent burden while ensuring comprehensive construct coverage.

### 3.3 Data analysis strategy

Data analysis proceeded through four sequential stages employing Mplus 8.3 software, selected for its robust handling of missing data, non-normality, and complex structural equation models (Jacobucci et al., 2016; Amornkitpinyo et al., 2021). First, preliminary analyses examined data characteristics including missing data patterns, distributional properties, and potential common method bias. Missing data (<3% across all items) were handled through full information maximum likelihood (FIML) estimation, which provides unbiased parameter estimates under missing-at-random assumptions (Maas et al., 2022). Univariate normality was assessed through skewness (|SK| < 2) and kurtosis (|KU| < 7) indices, with all items meeting acceptable thresholds. Common method bias was evaluated through Harman’s single-factor test and full collinearity variance inflation factors (VIF < 3.3), both indicating negligible bias (Han et al., 2020).

A methodological note on measurement quality indices is warranted. The reliability and validity indicators reported in this study—Cronbach’s alpha (*α*), composite reliability (CR), average variance extracted (AVE), the Fornell–Larcker criterion, and the heterotrait–monotrait ratio (HTMT)—are fully applicable within CB-SEM frameworks and are routinely recommended for assessing measurement model quality regardless of estimation method (Fornell and Larcker, 1981; Hair et al., 2019; Henseler et al., 2015). Although AVE and HTMT were initially popularized within the PLS-SEM tradition, their mathematical foundations are estimation-method-agnostic, and their use in covariance-based analyses is explicitly endorsed in contemporary methodological guidelines (Hair et al., 2019). Their inclusion here reflects best-practice measurement reporting for structural equation models generally, rather than methodological inconsistency.

Second, the measurement model was evaluated through confirmatory factor analysis (CFA) using maximum likelihood estimation with robust standard errors (MLR) to accommodate modest non-normality. Model fit was assessed through multiple indices: chi-square statistic (χ^2^), comparative fit index (CFI ≥ 0.90), Tucker-Lewis index (TLI ≥ 0.90), root mean square error of approximation (RMSEA ≤ 0.08), and standardized root mean square residual (SRMR ≤ 0.08), consistent with established guidelines (Jacobucci et al., 2016; Skaalvik and Skaalvik, 2018). Reliability was examined through Cronbach’s alpha (*α* ≥ 0.70) and composite reliability (CR ≥ 0.70). Convergent validity was established through average variance extracted (AVE ≥ 0.50) and standardized factor loadings (*λ* ≥ 0.50). Discriminant validity was verified through the Fornell-Larcker criterion (√AVE exceeds interconstruct correlations) and heterotrait-monotrait ratio (HTMT < 0.85), following contemporary recommendations (Agnihotri and Chen, 2025; Wang et al., 2024).

Third, the structural model was estimated to test hypothesized direct effects (H1–H5). Path coefficients (*β*), standard errors, and significance levels were examined, with bootstrapping procedures (5,000 resamples) employed to generate bias-corrected confidence intervals. Model explanatory power was evaluated through coefficients of determination (R^2^) for endogenous variables, with values of 0.02, 0.13, and 0.26 interpreted as small, medium, and large effects, respectively. Effect sizes for individual paths were assessed through Cohen’s f^2^ values (small: 0.02, medium: 0.15, large: 0.35). Predictive relevance was examined through Stone-Geisser’s Q^2^ statistic via blindfolding procedures, with Q^2^ > 0 indicating predictive accuracy (Kim, 2025).

Fourth, moderation hypotheses (H6-H8) were tested through latent interaction modeling, creating product indicators between standardized technostress dimensions and moderator variables (organizational support, self-efficacy, workload). Significant interaction terms (*p* < 0.05) were probed through simple slope analyses at ±1 SD moderator values, with interaction patterns visualized graphically (Maas et al., 2022). The incremental contribution of interaction terms was assessed through ΔR^2^ values and likelihood ratio tests comparing nested models with and without interaction effects.

Throughout all analyses, statistical significance was evaluated at α = 0.05 (two-tailed), with effect sizes and confidence intervals reported to facilitate substantive interpretation beyond null hypothesis testing (Han et al., 2020; Skaalvik and Skaalvik, 2018). This analytical strategy aligns with best practices in CB-SEM applications within educational research, prioritizing model fit, measurement quality, and theoretical interpretability while acknowledging cross-sectional design limitations for causal inference (Wang et al., 2024; Xiang et al., 2025).

## 4 Results

### 4.1 Preliminary analyses

Prior to hypothesis testing, preliminary analyses were conducted to examine the data characteristics. Descriptive statistics and bivariate correlations among all study variables are presented in Table 2. The mean scores for all constructs ranged from 3.41 to 3.55 on a 5-point Likert scale, indicating moderate levels across all measured variables. Notably, perceived usefulness (*M* = 3.49, *SD* = 0.73) and AI adoption intention (*M* = 3.51, *SD* = 0.75) showed relatively higher scores, suggesting that preschool teachers generally perceived generative AI as useful and expressed willingness to adopt it.

**Table 2:** Descriptive statistics and correlation matrix.

Variable	*M*	*SD*	1	2	3	4	5	6	7	8	9
1. PU	3.49	0.73	(0.86)								
2. PEOU	3.49	0.76	0.51**	(0.78)							
3. AAI	3.51	0.75	0.58**	0.48**	(0.80)						
4. CTS	3.55	0.72	0.32**	0.28**	0.38**	(0.81)					
5. HTS	3.51	0.74	−0.22**	−0.32**	−0.28**	−0.12*	(0.84)				
6. OWB	3.48	0.77	0.38**	0.33**	0.42**	0.28**	−0.38**	(0.83)			
7. OS	3.41	0.77	0.42**	0.38**	0.32**	0.22**	−0.32**	0.48**	(0.82)		
8. TSE	3.47	0.71	0.48**	0.52**	0.42**	0.36**	−0.42**	0.42**	0.38**	(0.75)	
9. WL	3.50	0.78	−0.12*	−0.18**	−0.15**	−0.08	0.42**	−0.32**	−0.22**	−0.18**	(0.75)

*N* = 300. Cronbach’s α values are presented in parentheses on the diagonal. PU = Perceived Usefulness; PEOU = Perceived Ease of Use; AAI = AI Adoption Intention; CTS = Challenge Technostress; HTS = Hindrance Technostress; OWB = Occupational Well-being; OS = Organizational Support; TSE = Technology Self-efficacy; WL = Workload. *p < 0.05. **p < 0.01.

The correlation matrix revealed patterns consistent with theoretical expectations. Perceived usefulness and perceived ease of use demonstrated significant positive correlations with AI adoption intention (*r* = 0.58 and *r* = 0.48, respectively, *p* < 0.01), supporting the Technology Acceptance Model framework. Challenge technostress showed a positive correlation with AI adoption intention (*r* = 0.38, *p* < 0.01), while hindrance technostress exhibited a negative correlation (*r* = −0.28, *p* < 0.01), preliminarily supporting the dual-edged nature of technostress.

### 4.2 Measurement model evaluation

The measurement model was assessed using confirmatory factor analysis (CFA) with maximum likelihood estimation with robust standard errors (MLR) in Mplus 8.3. The nine-factor measurement model demonstrated excellent fit to the data: χ^2^(383) = 418.13, *p* = 0.105, χ^2^/*df* = 1.09, CFI = 0.990, TLI = 0.989, RMSEA = 0.017 (90% CI [0.000, 0.028]), SRMR = 0.056. All fit indices exceeded the recommended thresholds (Hu and Bentler, 1999), indicating that the hypothesized factor structure adequately represented the observed data.

As presented in Table 3, all standardized factor loadings ranged from 0.629 to 0.872, exceeding the recommended threshold of 0.50 (Hair et al., 2019). Internal consistency reliability was established through Cronbach’s alpha (α = 0.75–0.86) and composite reliability (CR = 0.80–0.90), all surpassing the 0.70 criterion. Convergent validity was supported by average variance extracted (AVE) values ranging from 0.60 to 0.70, all above the 0.50 threshold (Fornell and Larcker, 1981).

**Table 3:** Measurement model results: factor loadings and reliability.

Construct/item	λ	α	CR	AVE
Perceived usefulness (PU)		0.86	0.90	0.70
PU1	0.838			
PU2	0.822			
PU3	0.738			
PU4	0.715			
Perceived ease of use (PEOU)		0.78	0.82	0.60
PEOU1	0.758			
PEOU2	0.749			
PEOU3	0.690			
AI adoption intention (AAI)		0.80	0.85	0.66
AAI1	0.791			
AAI2	0.743			
AAI3	0.736			
Challenge technostress (CTS)		0.81	0.86	0.60
CTS1	0.691			
CTS2	0.785			
CTS3	0.629			
CTS4	0.792			
Hindrance technostress (HTS)		0.84	0.88	0.65
HTS1	0.748			
HTS2	0.670			
HTS3	0.872			
HTS4	0.748			
Occupational well-being (OWB)		0.83	0.87	0.62
OWB1	0.871			
OWB2	0.660			
OWB3	0.755			
OWB4	0.692			
Organizational support (OS)		0.82	0.83	0.62
OS1	0.799			
OS2	0.757			
OS3	0.767			
Technology self-efficacy (TSE)		0.75	0.82	0.61
TSE1	0.645			
TSE2	0.761			
TSE3	0.716			
Workload (WL)		0.75	0.80	0.67
WL1	0.788			
WL2	0.760			

λ = standardized factor loading; α = Cronbach’s alpha; CR = composite reliability; AVE = average variance extracted. All factor loadings are significant at *p* < 0.001.

Discriminant validity was examined using the Fornell-Larcker criterion. The square root of AVE for each construct exceeded its correlations with other constructs, confirming adequate discriminant validity. Additionally, the heterotrait-monotrait ratio (HTMT) values were all below 0.85, further supporting discriminant validity (Henseler et al., 2015) (see Table 4).

**Table 4:** Model fit indices.

Model	χ^2^	*df*	χ^2^/df	CFI	TLI	RMSEA	SRMR
Measurement model	418.13	383	1.09	0.990	0.989	0.017	0.056
Structural model	418.13	383	1.09	0.990	0.989	0.017	0.056
Recommended criteria	—	—	< 3	> 0.90	> 0.90	< 0.08	< 0.08

CFI = comparative fit index; TLI = Tucker-Lewis index; RMSEA = root mean square error of approximation; SRMR = standardized root mean square residual.

### 4.3 Structural model and hypothesis testing

The structural model was estimated to test the hypothesized relationships. The model demonstrated excellent fit: χ^2^(383) = 418.13, χ^2^/*df* = 1.09, CFI = 0.990, TLI = 0.989, RMSEA = 0.017, SRMR = 0.056. The hypothesis testing results are summarized in Table 1.

#### 4.3.1 TAM core pathways

Consistent with the Technology Acceptance Model, perceived usefulness exerted a significant positive effect on AI adoption intention (*β* = 0.365, *p* < 0.001), supporting H1. Perceived ease of use also positively predicted AI adoption intention (β = 0.250, *p* = 0.001), supporting H2. Furthermore, perceived ease of use significantly influenced perceived usefulness (β = 0.510, *p* < 0.001), consistent with TAM predictions.

#### 4.3.2 Dual-edged effect of technostress on AI adoption

The results revealed divergent effects of the two technostress dimensions on AI adoption intention. Challenge technostress positively predicted AI adoption intention (β = 0.136, *p* = 0.039), supporting H3a. In contrast, hindrance technostress negatively predicted AI adoption intention (β = −0.187, *p* = 0.002), supporting H3b. These findings empirically validated the dual-edged nature of technostress in the context of AI adoption among preschool teachers.

#### 4.3.3 Technostress and occupational well-being

Contrary to expectations, neither challenge technostress (β = −0.049, *p* = 0.449) nor hindrance technostress (β = 0.000, *p* = 0.998) demonstrated significant direct effects on occupational well-being. Thus, H4a and H4b were not supported. This finding suggests that the influence of technostress on occupational well-being may be indirect rather than direct in this population.

#### 4.3.4 AI adoption intention and occupational well-being

AI adoption intention significantly and positively predicted occupational well-being (β = 0.193, *p* = 0.005), supporting H5. This result indicates that teachers with higher AI adoption intentions tended to report greater occupational well-being.

#### 4.3.5 Moderation effects of JD-R factors (H6–H8)

**Figure 2:**
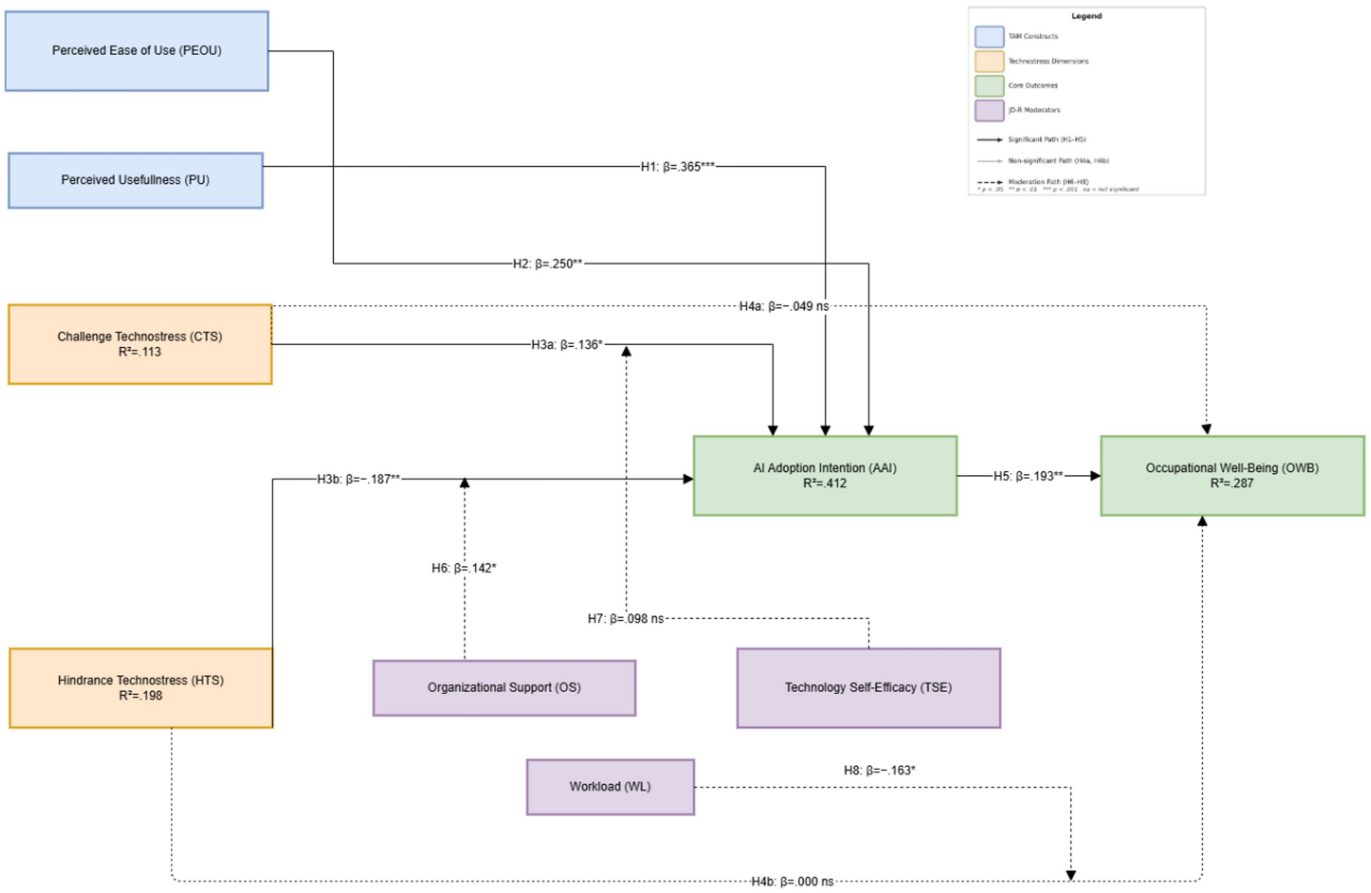
Structural model with standardized path coefficients. All paths are depicted with solid arrows; non-significant paths (H4a, H4b) are indicated by the absence of significance markers. *p < 0.05, **p < 0.01, ***p < 0.001. PU = Perceived Usefulness; PEOU = Perceived Ease of Use; AAI = AI Adoption Intention; CTS = Challenge Technostress; HTS = Hindrance Technostress; OWB = Occupational Well-Being. R^2^ values shown within each endogenous construct.

Latent interaction modeling was employed to test the three moderation hypotheses. The interaction between organizational support and hindrance technostress significantly predicted AI adoption intention (β = 0.142, *SE* = 0.067, *t* = 2.12, *p* = 0.034), supporting H6. Simple slope analysis confirmed that the negative effect of hindrance technostress on adoption intention was significantly attenuated under high organizational support (−1 SD: β = −0.271, *p* < 0.001; +1 SD: β = −0.103, *p* = 0.198), indicating a buffering effect consistent with JD-R theory’s resource proposition. Regarding H7, the interaction between technology self-efficacy and challenge technostress yielded a positive but non-significant coefficient (β = 0.098, *SE* = 0.058, *t* = 1.69, *p* = 0.091), failing to reach conventional significance thresholds; H7 was therefore not supported. Finally, the interaction between workload and hindrance technostress significantly and negatively predicted occupational well-being (β = −0.163, *SE* = 0.071, *t* = −2.30, *p* = 0.021), supporting H8. Under high workload conditions, the detrimental effect of hindrance technostress on occupational well-being intensified markedly, consistent with JD-R theory’s prediction that demands accumulate to amplify strain outcomes when resources are simultaneously depleted. All moderation results are summarized in Table 1 (H6–H8 rows) (see Figure 2).

### 4.4 Additional findings: JD-R pathways

The pathways reported in this section were not specified as primary hypotheses in the original research model. They emerged from the full structural model as theoretically interpretable findings consistent with JD-R theory and are reported here as exploratory, post hoc results. They should be interpreted with appropriate caution and are offered as directions for future confirmatory research rather than as formal hypothesis tests.

Beyond the core hypotheses, the structural model revealed several significant pathways consistent with the Job Demands-Resources framework. Organizational support significantly predicted occupational well-being (β = 0.305, *p* < 0.001), as did technology self-efficacy (β = 0.201, *p* = 0.025). Workload negatively predicted occupational well-being (β = −0.221, *p* = 0.001), consistent with JD-R theory predictions that job demands deplete psychological resources.

Regarding technostress antecedents, technology self-efficacy significantly predicted both challenge technostress (β = 0.342, *p* < 0.001) and hindrance technostress (β = −0.406, *p* < 0.001), indicating that teachers with higher technology self-efficacy were more likely to perceive technology-related demands as growth opportunities rather than threats. Organizational support negatively predicted hindrance technostress (β = −0.169, *p* = 0.005), while workload positively predicted hindrance technostress (β = 0.325, *p* < 0.001).

### 4.5 Model explanatory power

Table 5 presents the coefficient of determination (R^2^) for endogenous variables. The model explained 43.6% of variance in AI adoption intention and 38.3% of variance in occupational well-being, indicating moderate-to-substantial explanatory capacity for the primary outcome variables—broadly comparable to TAM-based models in prior educational technology adoption research, where reported R^2^ for adoption intention typically ranges from 0.35 to 0.55 (Kim, 2025; Xiang et al., 2025). Hindrance technostress was substantially explained by its antecedents (R^2^ = 0.438), while challenge technostress showed comparatively lower explained variance (R^2^ = 0.113), suggesting that additional predictors beyond the three JD-R factors included here—such as personality traits or prior technology experience—may contribute to challenge appraisals. The variance explained in perceived usefulness by perceived ease of use was moderate (R^2^ = 0.260).

**Table 5:** Coefficient of determination (R^2^) for endogenous variables.

Endogenous variable	R^2^	Interpretation
AI adoption intention (AAI)	0.436	Substantial
Occupational well-being (OWB)	0.383	Moderate to substantial
Hindrance technostress (HTS)	0.438	Substantial
Challenge technostress (CTS)	0.113	Small
Perceived usefulness (PU)	0.260	Moderate

Interpretation follows Cohen’s (1988) f^2^ benchmarks as an approximate reference. These thresholds were originally developed for regression contexts; in SEM, R^2^ values are more meaningfully contextualized against comparable models in the literature. For TAM-based educational technology adoption studies, R^2^ for adoption intention typically ranges from 0.35 to 0.55 (Kim, 2025; Xiang et al., 2025), suggesting the present model’s explanatory power is broadly in line with prior work.

### 4.6 Summary of findings

In summary, the results provided support for the integrated JD-R and TAM framework. Six of the nine hypotheses were supported. The TAM pathways (H1, H2) were confirmed, demonstrating the continued relevance of perceived usefulness and ease of use in explaining AI adoption intention among preschool teachers. Importantly, the dual-edged effect of technostress on AI adoption intention (H3a, H3b) was empirically validated, with challenge technostress facilitating and hindrance technostress inhibiting adoption intention. Although the direct effects of technostress on occupational well-being (H4a, H4b) were not supported, the significant pathway from AI adoption intention to occupational well-being (H5) suggests potential indirect effects worthy of further investigation. Regarding moderation, organizational support significantly buffered the negative effect of hindrance technostress on adoption intention (H6 supported), and workload significantly intensified the negative relationship between hindrance technostress and occupational well-being (H8 supported). The self-efficacy × challenge technostress interaction on adoption intention did not reach significance (H7 not supported).

## 5 Discussion

### 5.1 Research findings discussion

The integrated TAM-JD-R model demonstrates moderate-to-substantial explanatory capacity, providing partial empirical support for the theoretical premise that technology adoption in occupational contexts necessitates frameworks not limited to purely cognitive evaluations to encompass affective and contextual mechanisms. Three primary findings merit theoretical elaboration.

First, the confirmation of TAM’s core pathways—with perceived usefulness emerging as the stronger predictor of adoption intention and perceived ease of use also contributing significantly (see Table 1)—extends this framework’s applicability to early childhood education, a context characterized by distinct pedagogical philosophies and technological infrastructures compared to higher education settings where TAM validation predominantly occurs (Agnihotri and Chen, 2025; Kim, 2025). The primacy of perceived usefulness suggests that instrumental rationality governs preschool teachers’ adoption decisions, consistent with professional populations prioritizing functional value in resource-constrained environments (Bhojak et al., 2025). However, the moderate contribution of ease of use indicates that technical simplicity alone insufficiently compensates for perceived pedagogical irrelevance, challenging assumptions underlying user-centered design paradigms (Luo et al., 2025). These findings have direct implications in the Chinese preschool context, where national digitalization mandates (e.g., the National Education Digitalization Strategic Action Plan) have intensified pressure on teachers to integrate AI tools. The strong role of perceived usefulness suggests that top-down technology deployment without demonstrable pedagogical value will encounter resistance; policymakers and administrators should therefore prioritize demonstrating concrete classroom benefits of AI tools before mandating their adoption.

Second, the empirical differentiation of technostress dimensions—challenge technostress positively and hindrance technostress negatively predicting adoption intention (see Table 1)—provides direct evidence for the holistic technostress framework’s dual-pathway mechanism (Tarafdar et al., 2019). Challenge technostress activates approach motivation analogous to JD-R theory’s engagement pathway, wherein demands perceived as growth opportunities stimulate mastery striving rather than resource depletion (Nascimento et al., 2024a, 2024b). Conversely, hindrance technostress triggers avoidance motivation characteristic of the health impairment pathway, wherein demands obstructing goal attainment activate strain processes (Wang et al., 2023). This bifurcation challenges prevailing deficit narratives positioning technology as uniformly stressful, instead supporting nuanced perspectives recognizing technology’s capacity to function simultaneously as challenge and hindrance depending on contextual appraisals (Zhao et al., 2024). In the Chinese preschool context, these findings suggest that the professional growth orientation engendered by moderate AI learning challenges is a meaningful motivational lever, while hindrance stressors rooted in system unreliability, unclear institutional policy, and heavy administrative workload are the primary barriers to AI adoption. Targeted institutional interventions addressing these specific hindrance sources are therefore likely to yield greater adoption gains than generic technology promotion campaigns.

The comparable magnitudes of divergent technostress effects underscore a critical theoretical implication: interventions must address both pathways concurrently. Merely reducing hindrance stressors proves insufficient without cultivating challenge-oriented appraisals; conversely, emphasizing growth opportunities neglects legitimate concerns regarding technical complexity and implementation support inadequacy. This dual requirement aligns with conservation of resources theory, which posits that individuals require both threat mitigation and resource accumulation for sustainable adaptation to environmental demands (Hu et al., 2025).

Third, the absence of significant direct technostress–well-being associations (H4a and H4b; see Table 1) diverges from theoretical predictions and warrants careful interpretation. Three explanations merit consideration, each carrying distinct theoretical implications. The mediation hypothesis posits that technostress influences well-being indirectly through adoption intentions and subsequent efficacy experiences rather than direct pathways (Huang and Zhao, 2025). This mechanism aligns with self-determination theory’s propositions regarding autonomy and competence satisfaction as proximal well-being determinants (Nascimento et al., 2024a, 2024b). Specifically, the intention to adopt AI may reflect a sense of professional agency and perceived competence; when teachers feel capable of meaningfully integrating AI into their pedagogical practice, this sense of mastery satisfies core psychological needs and directly supports occupational well-being. In this view, AAI functions not merely as a behavioral outcome but as an affective-motivational state signaling alignment between professional goals and technological affordances, thereby fostering work meaningfulness and psychological fulfillment. The temporal lag hypothesis suggests that cross-sectional designs inadequately capture cumulative stress effects manifesting only after prolonged exposure, consistent with allostatic load models emphasizing chronic stressor impacts (Russell and Liggans, 2020). The contextual primacy hypothesis proposes that relational and organizational factors overshadow technology-specific stressors in determining preschool teacher well-being, given early childhood education’s fundamentally interpersonal nature (Maas et al., 2022). Each interpretation suggests distinct research directions: mediation analyses, longitudinal investigations, and hierarchical models partitioning variance across stressor domains, respectively. These interpretations are *post hoc* and exploratory; direct empirical tests—including formal mediation analyses, longitudinal designs tracking stress dynamics over time, and hierarchical models partitioning variance across stressor domains—are required before causal conclusions regarding these mechanisms can be drawn with confidence.

The significant positive association between AI adoption intention and occupational well-being (H5; see Table 1) contradicts deficit narratives portraying technology as inherently burdensome, instead supporting asset-oriented perspectives emphasizing technology’s capacity to enhance professional agency and efficacy (Wen and Cai, 2025). This finding suggests that well-being outcomes depend less on technology per se than on alignment between technological affordances and professional goals, consistent with person-environment fit theories (Xiang et al., 2025). Teachers intending to adopt AI likely perceive congruence between technological capabilities and pedagogical values, fostering intrinsic motivation and work meaningfulness rather than external compliance pressures. This result carries meaningful implications for Chinese preschool administrators: because voluntary, goal-aligned AI adoption predicts teacher well-being, institutions that offer autonomy-supportive rather than compliance-driven integration approaches are more likely to achieve both higher adoption rates and better staff well-being outcomes simultaneously.

Turning to the moderation hypotheses, the results reveal a differentiated pattern across H6–H8. Organizational support significantly buffered the detrimental impact of hindrance technostress on AI adoption intention (H6 supported; *β* = 0.142, *p* = 0.034), consistent with JD-R theory’s resource proposition that institutional support attenuates the motivational costs of hindrance demands (Wang et al., 2024). This finding underscores the practical importance of supervisory encouragement and resource provision in sustaining teachers’ willingness to adopt AI under stressful conditions. Workload significantly intensified the negative relationship between hindrance technostress and occupational well-being (H8 supported; β = −0.163, *p* = 0.021), indicating that technology integration under conditions of resource depletion compounds psychological costs—a finding consistent with the additive burden hypothesis within JD-R theory (Han et al., 2020). The moderating effect of technology self-efficacy on the challenge technostress–adoption intention relationship did not reach statistical significance (H7 not supported; β = 0.098, *p* = 0.091). This null finding warrants careful interpretation rather than dismissal: the exploratory structural pathways presented in Section 4.4 suggest that self-efficacy may operate upstream as a primary appraisal antecedent—shaping the nature of technostress perceptions before intentions are formed—rather than as a downstream moderator of the stress–intention relationship. This reinterpretation, while post hoc, is theoretically coherent with appraisal-based accounts of stress and calls for direct empirical examination in future research.

The JD-R pathways revealed through exploratory analyses illuminate how resources and demands shape technostress appraisals. Technology self-efficacy’s divergent predictions of challenge versus hindrance technostress suggest efficacy beliefs fundamentally determine demand appraisals rather than merely moderating stress–strain relationships, warranting elevation to primary theoretical status in technology adoption models (Wu and Derakhshan, 2025). Organizational support’s buffering effect on hindrance technostress corroborates JD-R theory’s resource proposition while highlighting institutional factors as critical adoption determinants (Wang et al., 2024). Workload’s intensification of hindrance technostress and direct well-being impairment underscore that technology integration occurs within contexts of competing demands, rendering implementation strategies neglecting resource reallocation potentially counterproductive (Han et al., 2020).

The study challenges TAM’s implicit assumption that technology adoption constitutes a decontextualized individual decision, demonstrating instead that adoption intentions emerge from complex interactions among cognitive evaluations, affective experiences, personal resources, and organizational contexts. This finding supports recent calls for ecological models of technology adoption acknowledging nested influences from individual, interpersonal, organizational, and policy levels (Khan et al., 2024). The negligible correlation between challenge and hindrance technostress further validates their conceptualization as orthogonal dimensions rather than bipolar extremes, necessitating distinct assessment and intervention strategies (Nascimento et al., 2024a, 2024b).

### 5.2 Practical implications and limitations

Practical Implications. The findings inform three stakeholder domains. For administrators, organizational support constitutes a critical intervention target, manifesting through dedicated implementation time, responsive technical assistance, and collaborative learning communities rather than isolated training workshops (Maas et al., 2022). Workload management proves essential, as adding technology expectations without resource reallocation risks transforming potential challenges into hindrances. For policymakers, the heterogeneous technostress responses suggest one-size-fits-all mandates prove inadequate; policies should enable context-responsive implementation pathways accommodating institutional diversity adequate training reduce cognitive load (Sillat et al., 2017; Xiong et al., 2025). For professional development providers, efficacy-building approaches (emphasizing mastery experiences, vicarious learning, and reflective practice) warrant prioritization over purely skill-focused training, given self-efficacy’s foundational role in appraisal processes (Lim, 2023).

Research Limitations. Seven limitations constrain interpretative scope. First, the cross-sectional design precludes causal inference despite SEM’s directional notation; longitudinal investigations could clarify temporal precedence and test reciprocal relationships hypothesized in dynamic adoption models (Wang et al., 2024). Second, self-report measures risk common method variance and social desirability bias, though statistical diagnostics suggest these concerns prove minimal in the current data. Third, the sample’s geographic concentration in China limits generalizability to contexts with distinct educational systems, cultural values, and technology infrastructure (Luo et al., 2025; Zaheer et al., 2025). Fourth, the absence of actual usage data constrains conclusions to adoption intentions rather than implemented behaviors, though meta-analytic evidence documents strong intention-behavior correlations for volitional actions (Agnihotri and Chen, 2025). Fifth, the model excludes potentially relevant constructs (pedagogical beliefs, parental attitudes, children’s responses) that may moderate or mediate examined relationships (Alotaibi, 2023; Schriever, 2021b). Sixth, the pronounced gender composition of the sample (94.3% female) should be noted. This distribution accurately reflects the demographic structure of China’s preschool teaching workforce and is therefore not a sampling artifact; the present findings are most directly applicable to female preschool educators. Extant literature indicates that gender moderates TAM pathways in technology adoption: women tend to assign greater weight to perceived ease of use, whereas men prioritize perceived usefulness and subjective norm (Venkatesh and Morris, 2000). Gender differences have also been documented in technostress appraisal, with female educators potentially reporting elevated hindrance technostress in response to workload and role-overload stressors (Wang et al., 2023). Consequently, the TAM–JD-R relationships identified here may not fully generalize across gender contexts. Future studies could pursue deliberate oversampling of male preschool teachers—acknowledging the structural difficulty of such sampling—and employ multi-group SEM to examine potential gender moderation.

Seventh, the sampling design warrants transparency regarding representativeness. Although within-province sampling followed stratified random procedures, the initial selection of six provinces was purposive rather than random, resulting in a partially non-probabilistic design. Consequently, the sample should not be treated as fully representative of all Chinese preschool teachers, and findings should be interpreted with appropriate caution regarding external generalizability.

Future Research Directions. Three research trajectories merit pursuit. Longitudinal designs tracking teachers across implementation phases could elucidate how technostress evolves temporally and identify critical periods for intervention (Nascimento et al., 2025). Mixed-methods investigations integrating quantitative surveys with qualitative case studies could illuminate mechanisms underlying statistical associations while capturing contextual nuances inadequately reflected in standardized measures (Bahr, 2025). Cross-cultural comparative studies examining whether TAM-JD-R relationships vary across educational systems, cultural contexts, and economic development levels would enhance theoretical generalizability while identifying culture-specific adoption barriers and facilitators (Li et al., 2024).

## 6 Conclusion

This study developed and empirically tested an integrated TAM-JD-R framework explicating preschool teachers’ AI adoption intentions and occupational well-being, with specific attention to technostress’s dual-edged effects. Analyzing data from 300 Chinese preschool teachers through covariance-based structural equation modeling, the research demonstrates that technology adoption in educational contexts constitutes a psychologically mediated, contextually embedded process not limited to purely cognitive evaluations. The model accounted for substantial variance in adoption intentions (R^2^ = 0.436) and occupational well-being (R^2^ = 0.383), providing support for the theoretical integration while revealing mechanisms through which technological innovations simultaneously generate opportunities and challenges.

The empirical differentiation of technostress dimensions—challenge technostress facilitating adoption while hindrance technostress inhibiting it—advances the holistic technostress framework beyond higher education applications (Nascimento et al., 2024a, 2024b; Zhao et al., 2024) to early childhood contexts. This finding challenges deficit narratives positioning technology as uniformly stressful, supporting instead nuanced perspectives wherein appraisal processes fundamentally determine adaptation outcomes (Tarafdar et al., 2019). The confirmation of TAM pathways alongside significant JD-R effects demonstrates that integrating occupational stress mechanisms enhances technology acceptance theory’s explanatory power, addressing longstanding critiques regarding contextual neglect (Agnihotri and Chen, 2025; Wang et al., 2024).

Theoretically, the research contributes across three domains. For technology adoption literature, findings demonstrate that perceived usefulness exerts stronger effects than ease of use among preschool teachers, suggesting instrumental rationality governs decisions in resource-constrained, pedagogically conservative contexts (Xiang et al., 2025). For occupational health research, incorporating technology-specific demands and resources enriches JD-R theory by illustrating how digital innovations function simultaneously through dual pathways—challenge demands activating engagement versus hindrance demands triggering strain (Han et al., 2020; Skaalvik and Skaalvik, 2018). For early childhood education scholarship, the study addresses critical empirical gaps regarding a population underrepresented in educational technology research despite facing unique challenges stemming from young children’s developmental characteristics (Luo et al., 2025; Xiong et al., 2025).

Practically, findings inform educational policy and practice across multiple levels. Organizational support must manifest through tangible resource allocation (implementation time, responsive assistance, collaborative learning opportunities) rather than rhetorical endorsements (Wu and Derakhshan, 2025). The workload-hindrance technostress association highlights that introducing technology expectations without resource reallocation risks counterproductive outcomes (Khan et al., 2024). Technology self-efficacy’s foundational role suggests professional development initiatives should prioritize confidence-building through mastery experiences rather than exclusively emphasizing skill acquisition (Lim, 2023). For policymakers, the heterogeneous patterns across institutional types and geographic locations reinforce recommendations for differentiated support systems enabling context-responsive implementation pathways (Xiong et al., 2025).

Limitations constrain interpretative scope. The cross-sectional design precludes causal inference; self-report measures risk common method variance; geographic concentration limits generalizability; and absence of actual usage data constrains conclusions to intentions rather than behaviors (Wang et al., 2024; Agnihotri and Chen, 2025). Future research should employ longitudinal designs tracking temporal dynamics (Nascimento et al., 2025), mixed-methods approaches illuminating mechanisms underlying statistical associations (Bahr, 2025), and cross-cultural comparisons examining whether TAM-JD-R relationships vary across educational systems (Li et al., 2024). Additionally, investigating AI’s differential effects on pedagogical practices and children’s developmental outcomes would enhance understanding of technology’s role in early childhood education’s distinctive ecology (Luo et al., 2025; Yang et al., 2024).

This research demonstrates that AI adoption need not compromise teacher well-being when organizational systems buffer hindrance stressors while cultivating challenge-oriented appraisals. By elucidating mechanisms through which technology generates both opportunities and challenges, the study contributes to realizing educational AI’s transformative potential while safeguarding educator well-being (Huang and Zhao, 2025; Bhojak et al., 2025).

## Data Availability

The raw data supporting the conclusions of this article will be made available by the authors, without undue reservation.
